# Ultrasound-assisted pH shifting modification of *Euryale ferox* protein: physicochemical, structural and functional properties

**DOI:** 10.1016/j.ultsonch.2026.107998

**Published:** 2026-08-07

**Authors:** Zimeng Wang, Renyuan Tian, Ligong Zhai, Yanyan Zhao, Mingyuan Huang, Haibo Zheng, Guoyuan Xiong, Shengming Zhao

**Affiliations:** a School of Food Science and Engineering, Anhui Science and Technology University, No.999 Wenhui Road, Chuzhou 239000, PR China; b Anhui Province Key Laboratory of Functional Agriculture and Functional Food, Anhui Science and Technology University, Chuzhou 239000, PR China

**Keywords:** *Euryaleferox* protein, Sonication, pH shifting, Structure, Functional properties

## Abstract

The impacts of integrating ultrasonic treatment (560 W) with pH shifting at pH 2, 3, 7, 11, and 12 on the physicochemical, structural, and functional attributes of *Euryale ferox* protein were investigated. The results indicated that the ultrasound-assisted pH 11 modified *Euryale ferox* protein exhibited an increase in the absolute zeta potential from 5.802 mV (pH 7 control) to 9.283 mV, solubility from 17.88% to 50.68%, surface hydrophobicity from 22.27 μg to 40.87 μg, foaming capacity from 52.78% to 70.55%, and emulsifying activity from 10.81 m^2^/g to 13.69 m^2^/g, along with the maximum UV absorption peak intensity. Meanwhile, the particle size, turbidity, and intrinsic fluorescence intensity of *Euryale ferox* protein under this condition were reduced. FT-IR profiling provided evidence that the combined treatment altered the secondary structure of *Euryale ferox* protein, as evidenced by increased β-sheet and random coil contents alongside decreased α-helix and β-turn fractions. Rheological results demonstrated that the combined treatment reduced both the shear stress and apparent viscosity of *Euryale ferox* protein. Therefore, ultrasound combined with pH shifting modification conferred considerable improvements upon the functional characteristics of *Euryale ferox* protein by inducing structural modifications, thereby enhancing its prospective applications in food processing.

## 1 Introduction

*Euryale ferox*, commonly known as gorgon nut, is an annual aquatic plant belonging to the family Nymphaeaceae and genus Euryale. It is predominantly distributed in temperate and subtropical zones of East Asia, South Asia, and Southeast Asia. In China, it is primarily cultivated in Anhui and Jiangsu provinces [16]. *Euryale ferox* exhibits a notable abundance of protein, essential amino acids, mineral elements including calcium and phosphorus, and polyphenolic antioxidant compounds. It serves both medicinal and edible purposes, exhibiting antioxidant and anti-aging effects, with demonstrated benefits for cardiovascular and cerebrovascular diseases, kidney diseases, and chronic enteritis. Consequently, it is thus acclaimed as the “aquatic longan” or “aquatic ginseng” [32]. The crude protein content of *Euryale ferox* ranges from approximately 8% to 12%, exceeding that of cereal crops such as rice, maize, and sorghum. Its protein primarily comprises glutelin, albumin, globulin, and prolamin [14]. *Euryale ferox* protein contains a diverse array of amino acids with a total content of approximately 39.9%, surpassing the standard essential amino acid content (27%). It contains six essential amino acids required for adults as well as two additional essential amino acids for infants (histidine and arginine), indicating that *Euryale ferox* protein is a high-quality plant protein source [19]. Although *Euryale ferox* protein exhibits a harmoniously proportioned array of amino acid composition and high protein content, its functional properties, including emulsifying activity, foaming capacity, and solubility, are relatively poor, thereby failing to fully match the performance expectations of food processing. Therefore, modification is essential to boost its functional performance and diversify its roles in food manufacturing. Previous studies have demonstrated that appropriate physical, chemical, and biological modification treatments can further improve protein functional properties by altering protein structure. For instance, ultrasound [27], high-pressure homogenization [11], thermal processing [1], pH shifting [33], and enzymatic modification [20] have been adopted for the purpose of modifying protein architecture and functional attributes.

The pH-shifting method represents a widely adopted chemical strategy for modulating protein functionality. This approach involves transient exposure of proteins to extreme pH environments (acidic or alkaline), followed by restoration to neutral pH. Such treatment triggers conformational transitions—specifically, unfolding at non-physiological pH values and subsequent reassembly into molten globule configurations upon pH neutralization [5]. Concurrently, extreme pH conditions promote subunit dissociation and weaken hydrophobic associations, yielding a more relaxed tertiary architecture with enhanced functional attributes [36]. Previous studies have demonstrated that pH shifting markedly enhances the functional performance of plant proteins, exemplified by peanut protein [15], mulberry leaf protein Li et al. [23], Li et al. [25], and yellow field pea protein [31]. However, excessive pH shifting may induce pronounced denaturation of proteins accompanied by architectural disruption. Therefore, it is crucial to advance integrated modification techniques that combine pH shifting with other treatments to enhance protein functional properties while minimizing adverse effects.

Ultrasonic processing, as a novel non-thermal physical modality, has gained considerable traction in food engineering owing to its operational simplicity, ecological compatibility, energy economy, and processing efficiency. Current utilization spans emulsification, extraction, and structural/functional modification of proteins derived from both animal and botanical sources [13]. The underlying mechanism centers on acoustic cavitation—specifically, the nucleation, growth, and implosive collapse of microbubbles within the liquid medium. This phenomenon generates intense localized mechanical forces, including high-velocity shear, transient pressure spikes, and turbulent flow [12]. Beyond mechanical effects, sonochemical reactions yield reactive oxygen species (including hydroxyl radicals and superoxide anions) capable of oxidatively modifying amino acid side chains and fragmenting polypeptide backbones, consequently promoting protein unfolding and conformational rearrangement. Simultaneously, ultrasound-mediated rupture of non-covalent interactions (hydrogen bonds, electrostatic forces, hydrophobic associations) and potential induction of intermolecular cross-linking constitute key pathways through which acoustic energy modulates protein physicochemical behavior and functional performance [21]. Synergistic ultrasound-pH shifting protocols have demonstrated efficacy in enhancing the techno-functional characteristics of diverse plant proteins, including rapeseed [24], Hericium erinaceus [8], and amaranth [6] preparations. Nevertheless, the specific impacts of this dual-modification approach on the physicochemical attributes, conformational features, and functional performance of *Euryale ferox* protein remain unexplored. Unlike many extensively investigated plant proteins, *Euryale ferox* protein exhibits a relatively compact molecular structure and strong aggregation tendency, which may restrict molecular flexibility, reduce the exposure of hydrophobic and polar functional groups, and consequently limit its solubility and interfacial properties. Since this protein is mainly composed of albumin and globulin fractions with complex intermolecular interactions, single physical or chemical modification strategies may not be sufficient to effectively induce structural rearrangement and functional improvement. Therefore, ultrasound-assisted pH shifting treatment, combining ultrasound-induced cavitation effects with pH-driven conformational transitions, may provide a synergistic strategy to disrupt aggregation, regulate protein conformation, and enhance the functional properties of *Euryale ferox* protein. This study aimed to elucidate how ultrasound-assisted pH shifting treatment regulates the structural rearrangement and aggregation behavior of *Euryale ferox* protein, and how these structural changes are associated with the improvement of its functional properties. The effects of ultrasound treatment (560 W) combined with different pH shifting conditions (pH 2, 3, 7, 11, and 12) on the physicochemical properties, conformational characteristics, and functional performance of *Euryale ferox* protein were systematically investigated to reveal the mechanism underlying this combined modification and provide a theoretical basis for the high-value utilization of *Euryale ferox* protein in food processing.

## 2 Materials and methods

### 2.1 Materials

The *Euryale ferox* seeds used in this study were provided by Anhui Tianchang Yilin Euryale ferox Aquatic Products Co., Ltd. (Chuzhou, China), and all other reagents were of analytical grade.

### 2.2 Extraction of *Euryale ferox* protein

Defatted *Euryale ferox* seeds were comminuted using a high-speed pulverizer and sieved through an 80-mesh screen to obtain fine powder. Protein extraction from this matrix was accomplished via isoelectric precipitation preceded by alkaline solubilization [7]. Specifically, the powdered material was dispersed in deionized water at a mass-to-volume ratio of 1:30 (g/mL), with the suspension pH elevated to 10.0 through dropwise addition of 1 mol/L NaOH. The mixture was continuously stirred in a water bath at 40℃ for 2 h to extract EFP. Following incubation, the sample was subjected to centrifugal separation at 4℃ and 8000 × g for 10 min. The harvested supernatant was readjusted to pH 4.5 by adding 1 mol/L HCl. Following a 30 min equilibration period, the sample underwent centrifugal fractionation at 5000 × g and 4℃ for 15 min, and the resulting precipitate represented the crude EFP. The isolated EFP was subjected to sequential rinsing with deionized water via centrifugal purification at 7000 × g and 4℃ for 15 min, executed in triplicate. Subsequent operations encompassed pH neutralization to 7, lyophilization, and preservation at 4℃. The protein purity was determined to be 88.25% by the Kjeldahl method.

### 2.3 Ultrasound combined with pH shifting treatment

The dual-modification protocol was adapted from Taheri et al. [37] with minor adaptations. EFP was first suspended in deionized water to achieve a final concentration of 5% (w/v), with continuous magnetic stirring (1 h) to ensure thorough dispersion. The pH was adjusted to 2, 3, 11, and 12 using 0.5 mol/L HCl or 0.5 mol/L NaOH, followed by standing at 25℃ for 1 h, then readjusted to 7 and equilibrated for 30 min to obtain pH shifting EFP solutions designated as pH 2, pH 3, pH 11, and pH 12. A control group was established and designated as pH 7. The pH-shifted samples (30 mL) were subsequently subjected to ultrasound treatment with an ultrasonic generator (JY 92-IIN, Scientz Co., Ltd., China) equipped with a 6 mm (diameter) probe at a frequency of 20 kHz for 15 min (2 s on and 2 s off) at 560 W. During sonication, the sample was maintained below 20 °C by circulating an ice-water mixture through the jacket of a double-walled beaker. The yielding samples designated as UpH 2, UpH 3, UpH 11, and UpH 12. The EFP sample treated with ultrasound only was designated as UpH 7. All treated EFP samples were lyophilized and maintained at 4℃ in a sealed container for subsequent analysis.

### 2.4 Particle size and zeta potential

EFP was dispersed in deionized water at a concentration of 1 mg/mL (pH 7.0), and the particle size distribution, average particle size, and zeta potential were measured using a nanoparticle size analyzer (Zetasizer Pro, Malvern, Austria) at 25℃ [56].

### 2.5 Solubility

The solubility of EFP was quantified employing the analytical procedure reported by Chen, Xu, and Zhou [2]. EFP was dispersed in deionized water to formulate suspensions at 10 mg/mL (pH 7.0) and subjected to centrifugation at 4℃ and 5000 × g for 15 min to collect the supernatant. The protein concentration in the supernatant was determined by the Biuret method. An aliquot of 1 mL supernatant was mixed with 4 mL of Biuret reagent and allowed to stand at room temperature for 30 min, after which the absorbance was determined at 540 nm. The protein concentration was quantified based on a BSA standard curve. The solubility was calculated using the equation that follows:

$$Solubility\left(\%\right)=\frac{Protein\;content\;of\;supernatant}{10\;mg/mL}\times 100\%$$

### 2.6 Turbidity

The turbidity of EFP was determined according to the method of Tahir et al. [38]. The EFP suspensions (1 mg/mL, pH 7.0) were prepared in deionized water, and the absorbance at 600 nm employing a visible spectrophotometer (722 N, Shanghai Jinghua, China).

### 2.7 Foaming properties

The foaming capacity (FC) and foaming stability (FS) of EFP were evaluated employing the analytical protocol established by Kang et al. [17]. Portions of 15 mL EFP suspensions (10 mg/mL, pH 7.0), prepared in deionized water, were subjected to homogenization employing a homogenizer (FJ200-S, Shanghai Lichen, China) operating at 16,000 r/min for 2 min, promptly decanted into a graduated cylinder for documentation of initial foam volume (V_0_), with residual foam volume (V_30_) documented following a 30 min equilibration period. FC and FS were assessed through application of the ensuing equations:

$$FC=\frac{V_{0}}{15}\times 100\%$$

$$FS=\frac{V_{30}}{V_{0}}\times 100\%$$

### 2.8 Emulsifying properties

Aliquots of 12 mL EFP suspensions (10 mg/mL, pH 7.0), prepared in deionized water, were emulsified with 4 mL of soybean oil via homogenization at 14,000 r/min for 2 min. Immediately, 50 μL of emulsion was mixed with 5 mL of 0.1% SDS, and the absorbance (A_0_) was measured at 500 nm, followed by standing for 30 min and measuring the absorbance (A_30_) using the same method [30]. The emulsifying activity index (EAI) and emulsion stability index (ESI) were calculated using the following equations:

$$EAI\;\left(m^{2}/g\right)=\frac{2\times 2.303\times A_{0}\times N}{C\times \varphi \times L\times 10000}$$

$$ESI\;\left(min\right)=\frac{A_{0}}{A_{0}-A_{30}}\times 30$$

wherein N denotes the dilution factor (100), C corresponds to the protein concentration (g/mL), φ represents the oil volume fraction (0.25), and L indicates the path length (1 cm).

### 2.9 Surface hydrophobicity

The surface hydrophobicity of EFP was quantified employing the analytical protocol established by Zhao et al. [52], Zhao et al. [53]. Portions of 2 mL EFP suspensions (5 mg/mL, pH 7.0) in deionized water were combined with 400 μL of 1 mg/mL bromophenol blue solution, agitated at 25℃ for 10 min, and subjected to centrifugation at 6000 × g for 15 min. The resulting suspensions were quantified the absorbance at 595 nm. The surface hydrophobicity was manifested as the quantity of bromophenol blue bound, computed via the subsequent mathematical expression:

$$BPBbound\left(\mu g\right)=400\times \frac{{(A}_{\mathrm{control}}-A_{\mathrm{sample}})}{A_{\mathrm{control}}}$$

### 2.10 Intrinsic fluorescence spectroscopy

The intrinsic fluorescence spectra of EFP were evaluated employing the analytical protocol established by Li et al. [23], Li et al. [25]. The EFP suspensions (0.1 mg/mL, pH7.0) in deionized water were quantified the fluorescence intensity employing a fluorescence spectrophotometer (F97 Pro, Shanghai Lengguang, China). Instrumental parameters were conducted using these settings: λex 280 nm, λem 300–450 nm, slit 10 nm, scan speed 1000 nm/min.

### 2.11 Ultraviolet (UV) spectroscopy

The UV spectra of EFP were determined according to the method of Zheng et al. [57]. The UV absorption spectra of EFP suspensions (0.2 mg/mL, pH 7.0), prepared in deionized water, were quantified employing a UV–visible spectrophotometer (UV-1800, Shimadzu, Suzhou, China) spanning 200 to 400 nm at a scanning rate of 50 nm/min and a resolution of 0.5 nm. The second-derivative UV spectra (d^2^A/dλ^2^) were calculated and analyzed using Origin 2021.

### 2.12 Fourier transform infrared spectroscopy (FT-IR)

The freeze-dried EFP powder was triturated with KBr (1:100) and pressed into a pellet. Spectral acquisition was conducted via a Fourier transform infrared spectrometer (TENSOR 27, Bruker, Bremen, Germany) operating at 4 cm^−1^ resolution across the wavenumber interval of 4000 to 400 cm^−1^, with 32 accumulations per specimen. The acquired datasets were subsequently processed through Ominic 8.0 and Peakfit 4.12 for elucidation of secondary structural alterations in EFP [56].

### 2.13 Rheological analysis

The rheological behavior of EFP was quantified employing the analytical protocol established by Kang et al. [18]. The 3 mL EFP suspensions (30 mg/mL) were positioned on a P60 parallel plate for measurement of shear stress and apparent viscosity employing a rotational rheometer (HAAKE MARS, Thermo Fisher Scientific, Karlsruhe, Germany) at 25℃, 0.1 Hz, and a shear rate of 0.01–1000 s^−1^.

### 2.14 Statistical analysis

All experiments were performed in triplicate using independently prepared samples. The datasets were statistically interpreted via SPSS 27.0, with outcomes presented as arithmetic mean ± standard deviation (SD). Intergroup variations were assessed via one-way analysis of variance (ANOVA) coupled with Duncan's multiple range test, with *P* < 0.05 deemed statistically meaningful.

## 3 Results and discussion

### 3.1 Particle size and zeta potential

**Fig. 1:**
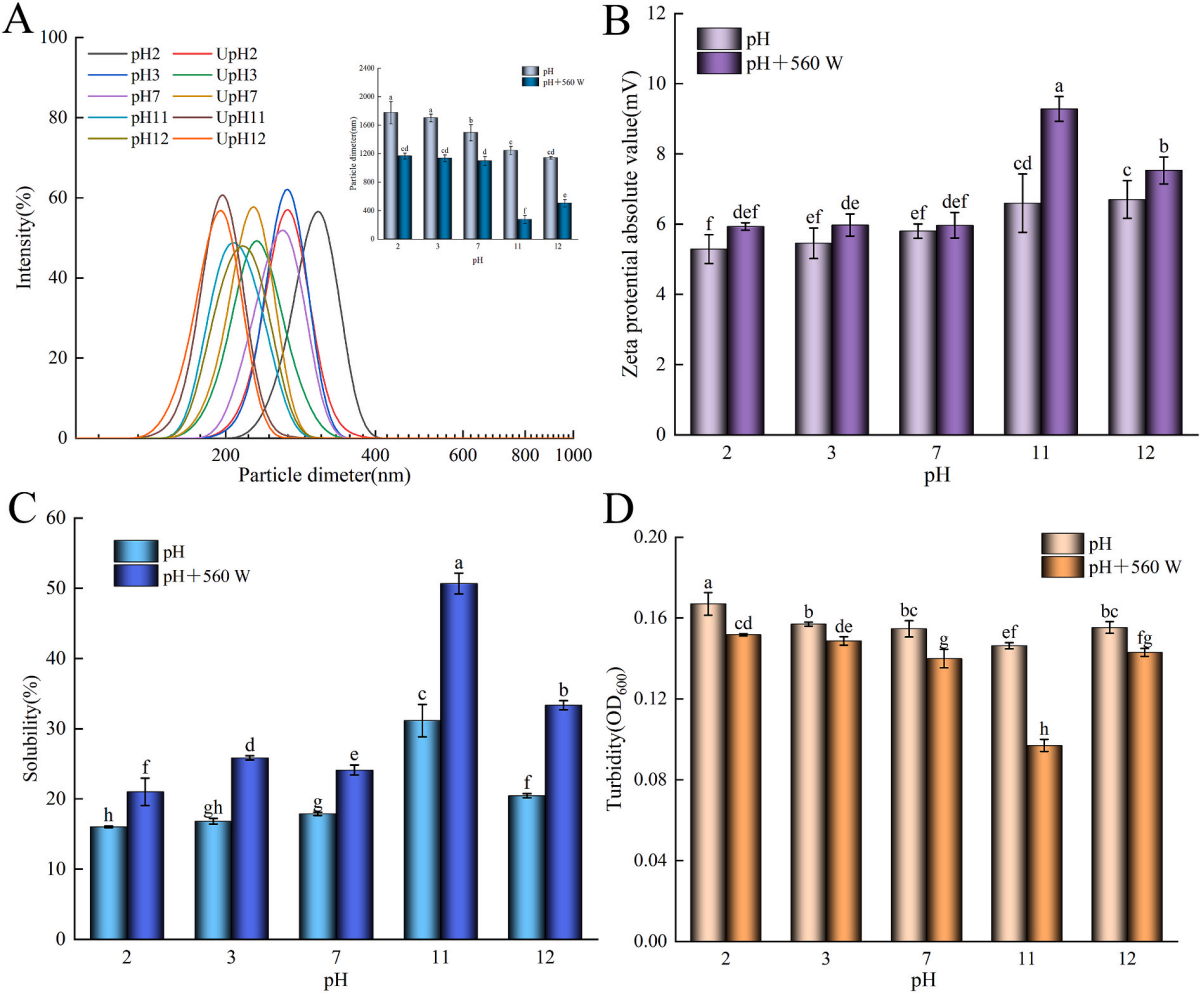
Effect of ultrasound combined with pH shifting on the particle size (A), absolute zeta potential value (B), solubility (C), and turbidity (D) of EFP. Different letters (a-h) indicate significant differences among groups (*P* < 0.05).

Dispersibility characteristics of EFP suspensions were first assessed through particle size distribution analysis (Fig. 1A). All modified samples, regardless of treatment regimen, displayed unimodal size distributions—an observation consistent with homogeneous particulate dispersion without apparent multimodal aggregation populations. Relative to the untreated control, acidic modification resulted in a pronounced rightward displacement of the size distribution maxima toward higher diameter values. This phenomenon may be associated with acid-induced changes in the charge state and intermolecular interactions of EFP molecules, which could promote protein association and aggregate formation under acidic conditions Kang et al. [18]. Conversely, alkaline treatment produced a distinct leftward shift toward finer particle dimensions. Such behavior may be attributed to the deviation of pH from the isoelectric point of EFP under alkaline conditions, which altered the surface charge characteristics and intermolecular interactions of protein molecules. Additionally, alkaline-induced conformational rearrangement may weaken protein aggregation and promote the dissociation of larger aggregates, thereby shifting the particle size distribution toward smaller particle sizes [10]. Meanwhile, the particle size distribution peak of EFP treated with ultrasound (560 W) shifted further toward smaller sizes, indicating further dispersion of EFP aggregates. This result is consistent with the reported effects of ultrasound and pH treatment on the particle size distribution of soy and potato proteins [35]. The influence of ultrasound-assisted pH shifting modification upon the average particle diameter of EFP suspensions is presented in Fig. 1A. The increased particle size observed under acidic conditions may be related to acid-induced conformational changes and exposure of previously buried hydrophobic regions, which may enhance protein–protein association and promote aggregate formation. Conversely, alkaline treatment markedly decreased the average particle size of EFP suspensions. This effect may be attributed to the increased electrostatic repulsion caused by the deviation from the isoelectric point, together with pH-induced structural rearrangement and ultrasound-mediated disruption of protein aggregates, which collectively reduced particle aggregation [50]. Furthermore, additional ultrasound treatment (560 W) intensified this effect. The average particle size of EFP underwent a marked contraction at pH 11 following ultrasonication, plummeting from 1243 nm to 275 nm. The observed size diminution likely reflects acoustic cavitation phenomena—specifically, the generation of intense hydrodynamic shear, localized pressure transients, and microstreaming turbulence that collectively overcome intermolecular cohesive forces, causing disintegration of supramolecular protein assemblies into finer constituents [38]. Similar trends were noted for sweet melon seed protein subjected to ultrasound combined with pH shifting. At pH 11, the protein particle size declined from 211.97 nm to 152.30 nm, demonstrating that this synergistic intervention markedly diminishes the mean particulate diameter [7].

Zeta potential serves as a quantitative proxy for the net surface charge density of colloidal protein particles, with its magnitude directly correlating with dispersion stability against flocculation [49]. The effect of ultrasound combined with pH-shifting treatment on the zeta potential of EFP is shown in Fig. 1B. The absolute zeta potential value of the control EFP was 5.802 mV. The absolute zeta potential values of EFP treated under acidic conditions showed no statistically significant deviation from the control sample, signifying that the net charge on the protein particle surface remained stable under acidic conditions without significantly altering the dissociation state of ionizable groups on the protein surface. In contrast, the absolute zeta potential value of EFP increased significantly under alkaline treatment conditions. It was further enhanced after ultrasound treatment (560 W), reaching a maximum of 9.283 mV under ultrasound combined with pH 11 treatment. This change may be associated with altered surface charge characteristics of EFP under alkaline conditions, coupled with the cavitation effect of ultrasound treatment may further induce partial structural rearrangement of proteins and expose polar groups, thereby contributing to changes in the surface charge distribution of protein particles [39]. The increased magnitude of zeta potential suggests enhanced electrostatic interactions among protein particles, which may partially contribute to changes in aggregation behavior. A higher absolute zeta potential value denotes greater electrostatic repulsion among protein particles, thereby further inhibiting particle aggregation and enhancing system stability. Similar changes in zeta potential induced by ultrasound combined with pH shifting induced comparable changes in the zeta potential of sweet melon seed protein isolate [48] and chickpea protein [43].

### 3.2 Solubility

Protein solubility constitutes a fundamental index of functional quality, exhibiting strong positive correlations with both emulsification capacity and foamability, and thus serving as a critical benchmark for assessing protein performance in food systems [51]. The impact of ultrasound combined with pH shifting treatment on the solubility of EFP is shown in Fig. 1C. The solubility of the control EFP was 17.88%. Compared with the control, the solubility of EFP gradually decreased under acidic conditions as the pH decreased, which may be attributed to the weakened intermolecular water associations and protein–protein interactions near the isoelectric point, altered protein surface charges, and reduced electrostatic repulsion among molecules promoting the assembly of large aggregates. Furthermore, the acidic milieu triggered structural rearrangements in polypeptides, resulting in the emergence of hydrophobic moieties and the subsequent self-assembly of macromolecules via hydrophobic associations, consequently diminishing aqueous dispersibility [7]. Under alkaline conditions, the solubility of EFP increased significantly, which presumably stems from the generation of abundant negative charges on the protein surface, resulting in strong intermolecular electrostatic repulsion that maintained proteins in a highly dispersed state. Additionally, the alkaline environment induced protein unfolding, exposing more hydrophilic and charged groups, thereby substantially enhancing hydration capacity and increasing solubility [42]. Ultrasound treatment (560 W) further enhanced EFP solubility, reaching a maximum of 50.68% at pH 11. The solubility enhancement primarily stems from sonomechanical effects: acoustic cavitation imposes sufficient mechanical stress to rupture multiple classes of non-covalent interactions (hydrogen bonding, hydrophobic association, and ionic pairing) within EFP suprastructures. This bond scission converts macroscopic insoluble aggregates into molecularly dispersed or finely particulate soluble species, concurrently expanding the protein-water interfacial area available for hydration, with net enhancement of aqueous solubility as the thermodynamic consequence Wang et al. [40], Wang et al. [41]. Additionally, the ultrasound-induced diminution in particle size and elevation in absolute zeta potential collectively suppressed further protein aggregation, which was also an important factor contributing to the enhanced solubility. Zhao et al. [54], Zhao et al. [55] also reported similar results, demonstrating that the physical forces generated by ultrasound could significantly improve the microstructure of walnut protein, yielding diminished particle dimensions and increasing the protein-water interfacial contact area, thereby enabling further solubility enhancement.

### 3.3 Turbidity

Turbidimetric analysis provides an indirect measure of protein aggregation propensity, wherein elevated optical density at 600 nm corresponds to increased light scattering by supramolecular protein clusters. The impact of ultrasound combined with pH shifting on EFP turbidity is visualized in Fig. 1D. Compared with the control (0.155), the turbidity of EFP significantly increased to 0.167 at pH 2. This may be explained by the emergence of hydrophobic groups arising from protein unfolding under strongly acidic conditions, consequently triggering protein aggregation. Additionally, the enlarged particle dimensions and diminished solubility of EFP under acidic conditions promoted the formation of numerous stable large aggregates, thereby elevating turbidity. Unlike the aforementioned, EFP showed substantially lower turbidity of 0.146 at pH 11, which may be attributed to the strong intermolecular repulsion generated under alkaline conditions, thereby reducing protein aggregation and lowering the turbidity. Following ultrasound treatment (560 W), the turbidity of EFP further decreased, reaching a minimum of 0.097 at UpH 11, congruent with the observed alterations in particle size (Fig. 1A) and zeta potential (Fig. 1B). This probably stems from ultrasound-driven cavitation and the accompanying high-speed shear forces, which dispersed large EFP aggregates into smaller particles, potentially altering surface charge characteristics and modulating protein–protein interactions, thereby enhancing dispersion uniformity and reducing the turbidity of EFP [4]. Zhang et al. [50] reported that the turbidity of UP-PPI was significantly reduced under pH 11–7 modification conditions, with peanut protein forming smaller aggregates in water and producing a clear and transparent solution.

### 3.4 FC and FS

**Fig. 2:**
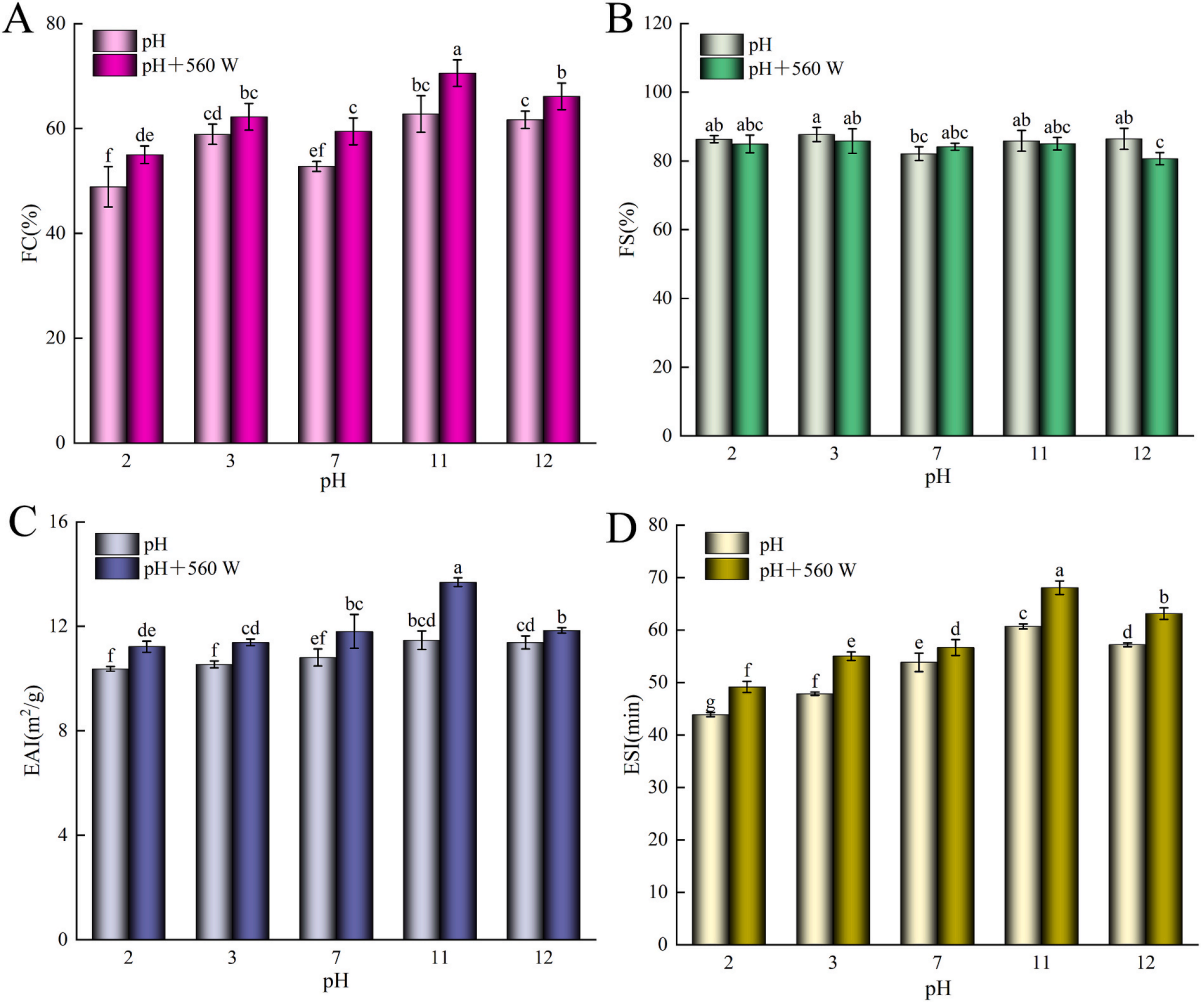
Effect of ultrasound combined with pH shifting on the foaming capacity (A), foaming stability (B), emulsifying activity (C), and emulsion stability index (D) of EFP. Different letters (a-f) indicate significant differences among groups (*P* < 0.05).

Foamability quantifies the propensity of proteins to generate and stabilize gas–liquid interfacial films—a surface-active behavior governed by the amphiphilic nature of protein molecules and their kinetic ability to undergo rapid conformational rearrangement at the air–aqueous boundary. The impact of ultrasound combined with pH-shifting on the FC and FS of EFP is presented in Fig. 2A. EFP displayed minimal FC (48.89%) at pH 2, in contrast to 52.78% observed for the untreated control, which is presumably caused by the weakened intermolecular electrostatic repulsion and reduced solubility under acidic conditions, hindering effective interfacial adsorption at the air–water boundary and resulting in decreased FC. At pH 11, the FC of EFP increased to 62.78%, indicating that extreme alkaline pH-shifting markedly improves the foaming ability of proteins. This is presumably caused by the partial unfolding of EFP under extreme alkaline conditions, followed by partial refolding upon readjustment to neutrality, giving rise to a molten globule state Wang et al. [40], Wang et al. [41]. Meanwhile, the reduced particle size and increased solubility of EFP under alkaline conditions further accelerated the migration of proteins toward the air–water interface, thereby enhancing the FC of EFP. Ultrasound treatment (560 W) further intensified this change, with the FC reaching a maximum of 70.55% under UpH 11 conditions. This is presumably caused by cavitation and turbulence generated by ultrasound, leading to the exposure of internal hydrophobic groups on the protein surface, thereby enhancing surface hydrophobicity and facilitating protein adsorption at the interface, ultimately increasing FC [48]. The FS of EFP showed no significant change under different modification conditions (Fig. 2B). This may be attributed to the increase in foam size over time, during which some EFP molecules desorbed from the air–water interface and aggregated with other molecules to form aggregates, thereby reducing interfacial activity and resulting in no significant change in FS compared with the untreated group [45]. Although the rheological results demonstrated variations in both shear stress and apparent viscosity across all treatment groups, the reduced apparent viscosity may have facilitated faster liquid drainage within the foam lamellae, thereby weakening foam stability and counteracting the potential positive effects associated with improved protein dispersion and reduced particle size. Conversely, increased apparent viscosity may have promoted the formation of more cohesive interfacial films; however, this advantage may have been offset by changes in protein conformational flexibility and interfacial adsorption behavior. Corroborative evidence was provided by Wang et al. [43], who reported no significant difference in FS of chickpea protein under various treatment conditions.

### 3.5 EAI and ESI

Emulsification performance characterizes the capacity of proteins to facilitate the formation and kinetic stabilization of thermodynamically unfavorable oil-in-water dispersions. This functionality is conventionally quantified through two complementary metrics: the EAI, which captures the interfacial area stabilized per unit mass of protein, and the ESI, which represents the temporal persistence of the dispersed state against coalescence and creaming. EAI reflects the ability of proteins to rapidly adsorb at the oil–water interface and reduce interfacial tension, whereas ESI reflects the ability of proteins to remain at the oil–water interface during emulsion storage. Fig. 2C illustrates the impact of combined ultrasound and pH shifting on the EAI of EFP. The EAI of EFP treated under acidic conditions showed no significant difference deviation from the control (10.81 m^2^/g), which is presumably caused by the phenomenon that although reduced solubility and increased particle size under acidic conditions tended to decrease he amphiphilic adsorption potential at the oil–water boundary, the absence of a significant difference in zeta potential between acidic and neutral conditions resulted in comparable surface charge densities and intermolecular electrostatic repulsion at the oil–water interface, thereby yielding no significant difference in EAI. At pH 11, the EAI of EFP markedly rose to 11.47 m^2^/g. This phenomenon may be ascribed to elevated solubility and diminished particulate dimensions, which expedited the movement and interfacial accumulation of proteins at the oil–water boundary. Further ultrasound treatment (560 W) significantly enhanced the EAI of EFP, reaching a maximum of 13.69 m^2^/g at UpH 11. This likely arises from the mechanical shear forces engendered by ultrasound treatment further reducing the particle size of proteins, thereby decreasing the interfacial tension and enabling more EFP to be adsorbed at the oil–water interface, ultimately promoting the formation of an emulsion system with a stable interfacial film [7]. Meanwhile, ultrasound treatment induced further unfolding of the tertiary conformation of EFP, leading to solvent exposure of hydrophobic groups that were more readily adsorbed onto oil droplets and interacted with lipids, thereby enhancing EAI [47]. Yang et al. [46] also reported similar results, illustrating that sonication coupled with pH-shifting modification considerably elevated the EAI of perilla protein.

EFP exhibited inferior emulsion stability relative to the control when subjected to acidic treatment (Fig. 2D), presumably owing to the burial of hydrophobic groups under acidic conditions, resulting in fewer exposed hydrophobic residues at the oil–water interface and difficulty in forming a dense and continuous interfacial film, thereby leading to a decrease in ESI. At pH 11, the ESI of EFP significantly increased to 60.69 min, which may be attributed to the exposure of hydrophobic regions groups, contributing to the formation of a more cohesive interfacial layer on the oil droplet surface and thereby enhancing ESI. Following ultrasound treatment (560 W), the ESI of EFP further increased, reaching a maximum of 68.07 min at UpH 11. This phenomenon may be ascribed to the cavitation effect produced by sonication, which diminished the surface tension of proteins at the oil–water boundary and disintegrated extensive protein assemblies, consequently amplifying the emergence of hydrophobic moieties. Thus, protein accumulation at the oil–water boundary was promoted, ultimately elevating ESI Kang et al. [18].

### 3.6 Surface hydrophobicity

**Fig. 3:**
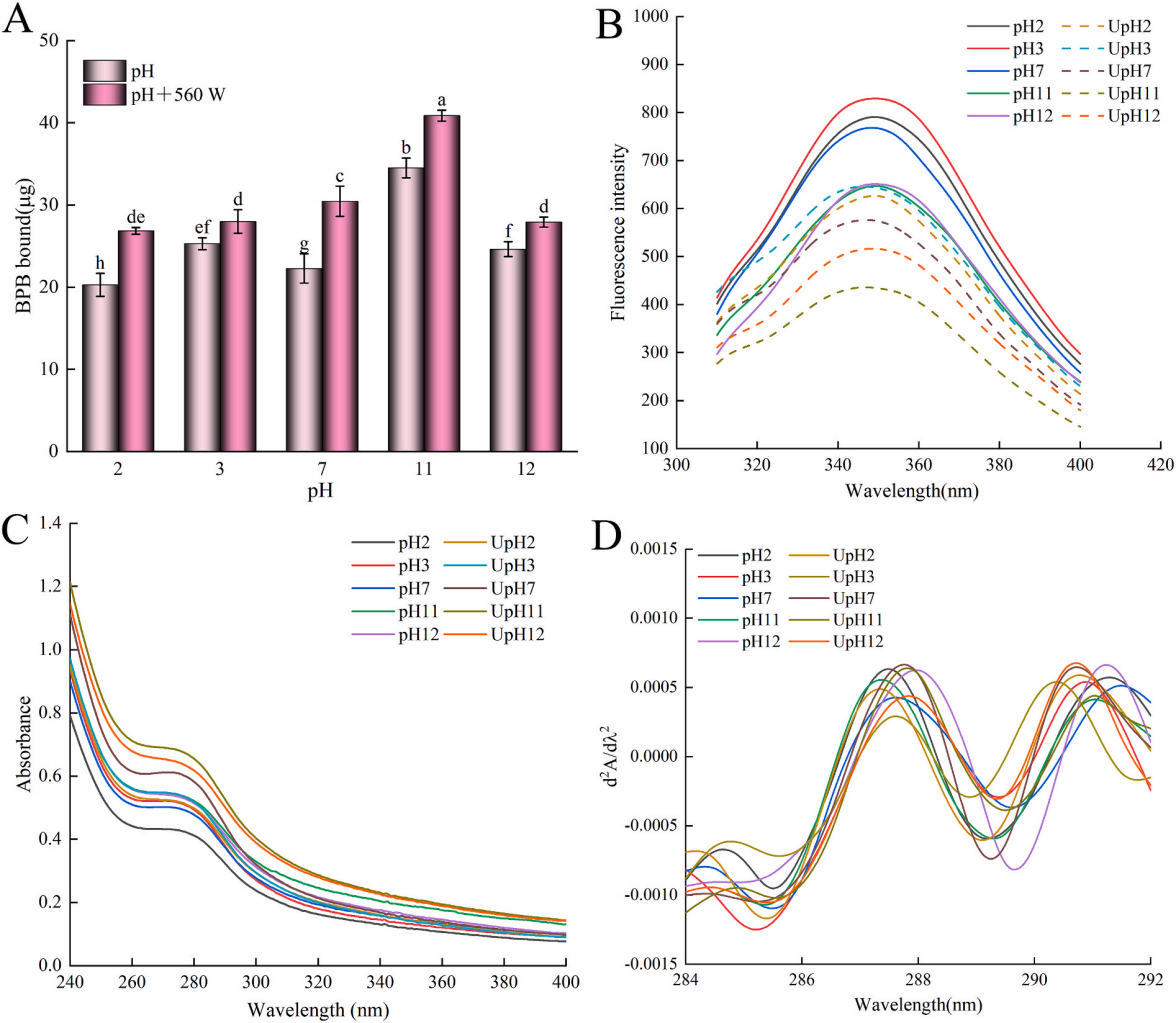
Effect of ultrasound combined with pH shifting on the surface hydrophobicity (A), intrinsic fluorescence spectra (B), UV spectra (C), and second derivative UV spectra (D) of EFP. Different letters (a-h) indicate significant differences among groups (*P* < 0.05).

Surface hydrophobicity, as quantified by chromophoric probe binding (bromophenol blue), reports on the spatial distribution and surface exposure extent of nonpolar amino acid side chains. This parameter bears direct relevance to interfacial functionalities (emulsification, foamability) and serves as a sensitive conformational probe for detecting tertiary structural perturbations. The impact of ultrasound combined with pH-shifting on the surface hydrophobicity of EFP is depicted in Fig. 3A. Compared with the control (22.27 μg), the surface hydrophobicity of EFP significantly decreased at pH 2, which may be explained by the excessive protein denaturation under extreme acidic conditions, driving the assembly of numerous insoluble aggregates and concomitant masking of hydrophobic groups, thereby decreasing surface hydrophobicity. Conversely, alkaline treatment elicited a marked elevation in protein surface hydrophobicity, peaking at 34.52 μg under pH 11 conditions. This likely stems from the profusion of negative charges decorating the protein surface under alkaline conditions, which enhanced intermolecular electrostatic repulsion and promoted unraveling of the protein tertiary structure. Consequently, hydrophobic amino acid residues originally buried inside were exposed on the molecular surface, thereby increasing the surface hydrophobicity [42]. Additional ultrasound treatment (560 W) led to a further increase in the surface hydrophobicity of EFP, peaking at 40.87 μg under UpH 11 conditions. This phenomenon may be ascribed to the acoustic cavitation induced by ultrasound, which diminished intermolecular forces and triggered protein structural unfolding, consequently facilitating the emergence of hydrophobic groups previously sequestered within the protein interior. Consequently, more bromophenol blue bound to the previously buried amino acid residues, thereby augmenting surface hydrophobicity [15]. Huang et al. [10] also reported similar findings, demonstrating that synergistic ultrasound and pH shifting treatment notably enhanced the surface hydrophobicity of yellow mealworm protein by inducing protein structural unfolding.

### 3.7 Intrinsic fluorescence spectroscopy

Intrinsic fluorescence measurements exploit the environmental sensitivity of aromatic chromophores (predominantly tryptophan) to monitor protein conformational dynamics. Specifically, shifts in emission maxima and quantum yield variations report on the polarity of the local dielectric environment surrounding these residues, thereby enabling detection of tertiary structural transitions involving burial or exposure of hydrophobic core regions. The impact of ultrasound combined with pH shifting on the intrinsic fluorescence intensity of EFP is illustrated in Fig. 3B. Relative to the control, acidic treatment increased the fluorescence intensity of EFP, likely reflecting pH-dependent conformational rearrangements that altered the microenvironment surrounding tryptophan residues. This increase in fluorescence intensity may indicate reduced fluorescence quenching due to changes in the accessibility and local environment of aromatic residues. Conversely, alkaline treatment decreased the fluorescence intensity of EFP, suggesting modifications to the tertiary structure and microenvironment of aromatic residues under alkaline conditions. This decrease may be ascribed to partial protein unfolding or rearrangement, which could alter the exposure and polarity of chromophore-containing regions, such as tryptophan residues. Consequently, the exposure of tryptophan residues from the protein interior enhanced fluorescence quenching, ultimately lowering the fluorescence intensity [43]. Ultrasound treatment (560 W) further reduced the fluorescence intensity of EFP, reaching a minimum under UpH 11 conditions. This likely arises from ultrasound-generated cavitation, which modifies the protein tertiary structure, facilitating the emergence of additional chromophores—including tryptophan—into the solvent milieu and eliciting fluorescence quenching, ultimately diminishing fluorescence intensity Zhao et al. [54], Zhao et al. [55]. Furthermore, ultrasound treatment modified the aggregation state of the protein, consequently altering the protein microenvironment and diminishing fluorescence intensity. Hong et al. [9] also reported similar results, demonstrating that modification treatment reduced the endogenous fluorescence intensity of *Spirulina platensis* protein, and that the synergistic application of 500 W ultrasonication and pH 12 substantially attenuated the intrinsic fluorescence intensity.

### 3.8 UV spectroscopy

Ultraviolet absorption spectroscopy in the 240–320 nm region captures electronic transitions within aromatic side chains (Trp, Tyr, Phe), with spectral position and intensity modulated by the local polarity, hydrogen bonding status, and solvent accessibility of these chromophores—making this technique valuable for tracking tertiary conformational changes. The impact of ultrasound combined with pH shifting on the UV spectra of EFP is presented in Fig. 3C. All treatment groups exhibited UV absorption peaks at 260–280 nm, primarily attributed to the conjugated π-electron systems in the aromatic ring side chains of tryptophan and tyrosine residues within the protein molecules, producing π–π* transitions upon UV excitation [3]. Acidic pH treatment drove down the UV absorption peak intensity of EFP compared with the control, which is likely due to the burial of aromatic amino acids within the molecule, resulting in reduced surface hydrophobicity and weakened UV absorption. Under alkaline conditions, the UV absorption peak intensity of EFP increased, indicating that the altered protein molecular structure resulted in chromophores previously sequestered within the protein interior becoming exposed to the solvent, thereby enhancing UV absorption intensity. Ultrasound treatment (560 W) further intensified this change, with the UV absorption peak intensity reaching a maximum at UpH 11. The underlying cause appears to be the implosive cavitation produced by ultrasound, which further unraveled the EFP structure and accelerated the chromophore exposure Li et al. [23], Li et al. [25]. Surface hydrophobicity and intrinsic fluorescence spectroscopy results also demonstrated that ultrasound coupled with alkaline pH shifting effectively induced protein structural unfolding, promoted hydrophobic group exposure, and facilitated the redistribution of internally sequestered hydrophobic amino acids toward the protein exterior, thereby altering the protein tertiary structure.

Due to the overlapping absorption peaks of different aromatic amino acids, it is difficult to effectively analyze the individual UV absorption changes. Therefore, second derivative UV spectroscopy was employed to effectively distinguish the microenvironmental changes of Try and Tyr and evaluate the degree of exposure [56]. The impact of ultrasound combined with pH shifting on the second derivative UV spectra of EFP is depicted in Fig. 3D. The second derivative UV spectra of EFP exhibited two peaks at 288 nm and 292 nm and two absorption minima at 285 nm and 290 nm spanning the 280–300 nm wavelength interval. The absorption maximum centered at 288 nm was primarily attributed to tryptophan and tyrosine residues, whereas the absorption maximum centered at 292 nm was exclusively assigned to tryptophan residues [44]. Relative to the control, both ultrasonication and alkaline treatment elicited a blue shift (toward shorter wavelengths) in the absorption peak positions of tryptophan and tyrosine residues of EFP, indicating that these residues migrated toward a more polar environment and were exposed from a hydrophobic to a hydrophilic environment. Second derivative UV spectroscopy observations corroborated those previously obtained by surface hydrophobicity (Fig. 3A) and intrinsic fluorescence spectroscopy (Fig. 3B), collectively demonstrating alterations in the tertiary structure of proteins.

### 3.9 FT-IR

**Fig. 4:**
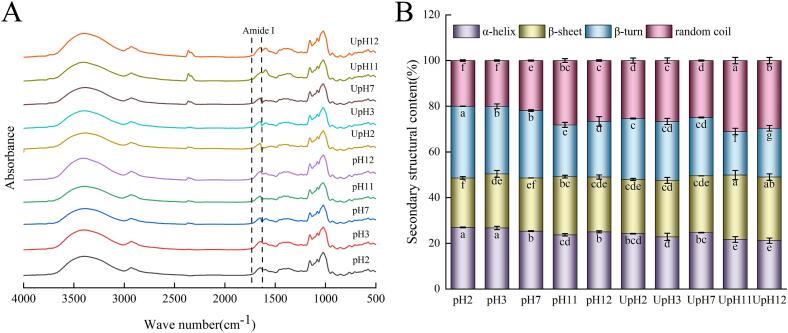
Effect of ultrasound combined with pH shifting treatment on the FTIR spectra (A) and secondary structure (B) of EFP. Different letters (a-g) indicate significant differences among groups (*P* < 0.05).

FT-IR was employed to monitor modifications in the secondary structural motifs of EFP following ultrasound-assisted pH shifting modification, with representative spectra presented in Fig. 4A. The amide A band located at 3200–––3400 cm^−1^ is primarily arises from N-H and O-H stretching vibrations in intermolecular hydrogen bonds, whereas the amide B band located at 2800–––3000 cm^−1^ corresponds to C-N stretching vibrations and asymmetric CH_2_ stretching vibrations [28]. The infrared spectral peak shapes of EFP treated with ultrasound combined with pH shifting were similar, indicating that no significant changes occurred in the protein backbone.

The amide I band at 1600–1700 cm^−1^ is a sensitive region reflecting changes in protein secondary structure, arising from C=O stretching vibrations. Changes in protein secondary structure are often analyzed by determining the proportions of α-helix (–1660 cm^−1^), β-sheet (1610–1640 cm^−1^ and 1670–1680 cm^−1^), β-turn (1660–1670 cm^−1^ and 1680–1700 cm^−1^), and random coil (1640–1650 cm^−1^) Zhao et al. [52], Zhao et al. [53]. As evidenced in Fig. 4B, decreases in both α-helix and β-turn percentages were observed for EFP following combined ultrasound and alkaline treatment relative to the control. In contrast, the percentages of β-sheet and random coil rose. This is presumably caused by the phenomenon that under alkaline conditions, protein molecules acquired a greater negative charge density, and the increased electrostatic repulsion promoted polypeptide chain unfolding, leading to partial disruption of α-helix regions. Concurrently, enhanced hydrophobic associations under alkaline conditions may have contributed to the diminished α-helix proportion and the amplified β-sheet proportion. After ultrasound treatment, the protein secondary structure was further unfolded, and large insoluble aggregates were transformed into smaller soluble aggregates, promoting the surface accessibility of both hydrophobic side chains and ionizable groups, thereby further leading to a loss of α-helical structure coupled with a gain in β-sheet content [56]. Random coil represents a completely loose peptide chain with no fixed spatial arrangement. After ultrasound treatment, numerous peptide chain hydrogen bonds and weak interactions were dissociated, large molecular aggregates were destroyed, the ordered protein structure was reduced, and random coil content increased. Sun et al. [35] reported that ultrasound, pH, and ultrasound combined with pH treatments all significantly reduced the contents of α-helix and β-turn in soy protein isolate and potato protein isolate, while significantly increasing random coil content, indicating that ultrasound combined with pH treatment could disrupt intermolecular interactions in proteins and thereby intensify structural disorder.

### 3.10 Static rheology

**Fig. 5:**
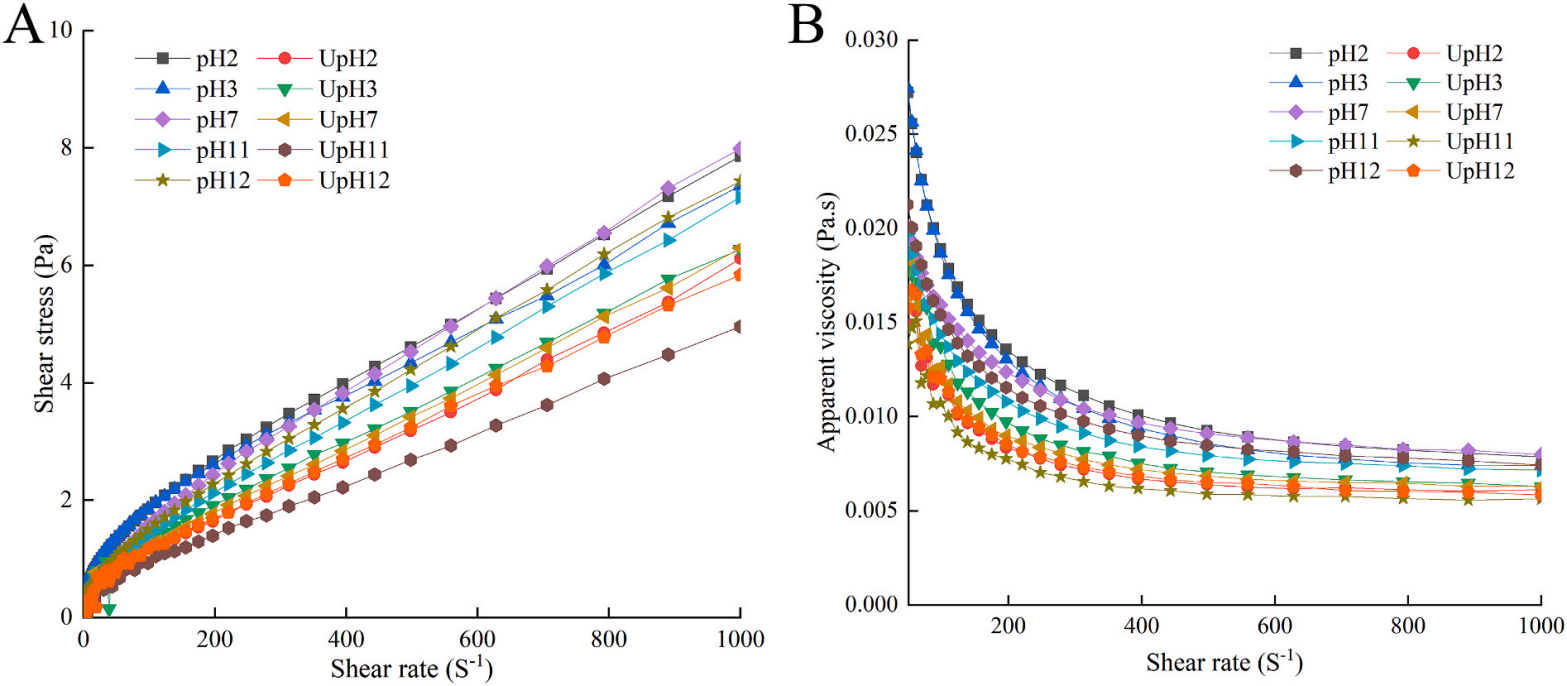
Effect of ultrasound combined with pH shifting treatment on the shear stress (A) and apparent viscosity (B) of EFP.

Steady-shear rheological measurements characterize the flow behavior of protein dispersions by quantifying the relationship between applied deformation rate (shear rate) and resulting resistive stress (shear stress), from which apparent viscosity is derived as the ratio of these quantities. Shear stress is an important indicator for evaluating protein stability and extensibility. The impact of ultrasound combined with pH shifting on the shear stress of EFP is depicted in Fig. 5A. Under the shear rate range of 0.01–––1000 s^−1^, the shear rate and shear stress of EFP exhibited a nonlinear increasing trend, characteristic of pseudoplastic fluid behavior. The shear stress of all EFP suspensions demonstrated a positive correlation with elevated shear rate. In the ultrasound-untreated group, the shear stress was lowest at pH 11 under identical shear rate, probably because alkaline conditions conferred abundant negative charges on proteins, disrupting the original intermolecular interaction network through electrostatic repulsion, inhibiting protein aggregation and thereby decreasing shear stress. After ultrasound treatment (560 W), the shear stress of EFP suspensions further decreased, reaching the minimum at UpH 11. This phenomenon may be ascribed to ultrasound-driven cavitation, which generated intensified shear forces that further compromised hydrogen bonding and hydrophobic associations, consequently inducing the unfolding of internal protein architecture and attenuating protein aggregation [34].

Apparent viscosity is commonly employed to evaluate protein structural changes and intermolecular interactions. The effect of ultrasound combined with pH shifting on the apparent viscosity of EFP is illustrated in Fig. 5B. With increasing shear rate, the apparent viscosity gradually decreased, exhibiting significant pseudoplastic flow behavior typical of non-Newtonian fluids. The observed shear-thinning response reflects the progressive dominance of hydrodynamic forces over Brownian randomization as the deformation rate increases. At elevated shear rates, EFP molecules undergo progressive orientation along streamlines, disrupting intermolecular entanglements and transient network structures, with consequent diminution of viscous resistance [22]. The apparent viscosity of EFP reached a minimum at UpH 11, presumably ascribable to the acoustic cavitation induced by sonication, which led to the cleavage of hydrogen bonds and disulfide bonds within protein molecules and the perturbation of hydrophobic associations [29]. Meanwhile, the occurrence of shear-thinning behavior was mechanistically connected to the functional performance of EFP, as smaller protein particle size and higher solubility could reduce interactions between proteins, enhance protein mobility, and thereby decrease apparent viscosity [26].

## 4 Conclusion

The present investigation establishes that synergistic ultrasound-pH shifting constitutes a potent approach for enhancing the techno-functional performance of *Euryale ferox* protein. Under extreme alkaline (pH 11) conditions, the particle size, turbidity, intrinsic fluorescence intensity, α-helix, β-turn, shear stress, and apparent viscosity of EFP decreased. Conversely, the absolute zeta potential value, solubility, surface hydrophobicity, foaming capacity, emulsifying activity, β-sheet content, random coil content, and UV absorption peak intensity significantly increased. The cavitation effect generated by ultrasound (560 W) further amplified these changes in the physicochemical, structural, and functional attributes of EFP. These findings provide a mechanistic rationale for deploying this green, non-thermal modification strategy to expand the food industry applications of underutilized aquatic plant proteins.

## CRediT authorship contribution statement

**Zimeng Wang:** Writing – original draft, Methodology. **Renyuan Tian:** Software, Methodology. **Ligong Zhai:** Resources. **Yanyan Zhao:** Supervision. **Mingyuan Huang:** Investigation. **Haibo Zheng:** Conceptualization. **Guoyuan Xiong:** Project administration, Funding acquisition. **Shengming Zhao:** Writing – review & editing, Funding acquisition.

## Declaration of competing interest

The authors declare that they have no known competing financial interests or personal relationships that could have appeared to influence the work reported in this paper.
